# Targeting Immune Checkpoint Proteins in Cancer Therapy and the Potential of RNAi-Based Immunotherapy

**DOI:** 10.3390/ph19081212

**Published:** 2026-08-01

**Authors:** Katherine Kaixin Wang, Ai-Ming Yu

**Affiliations:** Department of Biochemistry and Molecular Medicine, UC Davis School of Medicine, Sacramento, CA 95817, USA; kkawang@health.ucdavis.edu

**Keywords:** immune checkpoint proteins, immune checkpoint inhibition, cancer immunotherapy, RNA interference, microRNA, small interfering RNA

## Abstract

Cancer immunotherapy via targeting immune checkpoint proteins (ICPs) has transformed the treatment of multiple malignancies, offering improved clinical outcomes over conventional therapies. Immune checkpoint inhibitors (ICIs), including FDA-approved monoclonal antibodies against CTLA-4, PD-1, and PD-L1, as well as emerging agents targeting novel ICPs, have demonstrated strong therapeutic efficacy by restoring antitumor immune responses. In parallel, RNA interference (RNAi)-based approaches involving microRNAs (miRNAs) and small interfering RNAs (siRNAs) have emerged as promising alternative strategies for modulating ICP expression at the posttranscriptional level, enabling selective and simultaneous regulation of multiple immune checkpoint pathways. Preclinical and early clinical studies have indicated effective downregulation of target ICP expression and enhanced antitumor immunity across diverse cancer models. Due to the inherent instability of RNA molecules, the development of RNAi therapeutics has been accompanied by advances in delivery platforms. In this review, we discuss the biological functions of established and novel ICPs, along with immunotherapeutics approved by the FDA and under Phase III clinical development. We also provide an overview of the RNAi mechanism of miRNAs and siRNAs, highlight endogenous miRNAs that regulate immune checkpoint pathways, and summarize ICP-targeting RNAi agents and their corresponding delivery systems under preclinical and clinical development. Collectively, these advances underscore the potential of RNAi-based immune checkpoint modulation, complementing existing ICIs and expanding the next generation of cancer immunotherapy.

## 1. Introduction

Cancer immunotherapy has transformed the treatment landscape for several types of malignancies by producing durable clinical responses and improving survival in selected patient populations, especially those with melanoma and non-small cell lung cancer (NSCLC) [1,2]. While prior treatments, such as surgery, radiotherapy, chemotherapy, and targeted therapy, remain fundamental components of cancer management, immunotherapy has become an integral part of modern cancer treatment strategies, either as first-line or adjuvant therapy, for patients with advanced and metastatic cancer. Nevertheless, heterogeneous clinical responses and immune-related adverse events remain important challenges, driving the continued development of next-generation immunotherapeutic strategies [2].

Cancer immunotherapy is based on the concept of cancer immunoediting, a dynamic process in which the immune system both suppresses tumor development and shapes tumor evolution. Cancer immunoediting involves three phases, which are elimination (cancer immunosurveillance), equilibrium, and escape [3]. Although the immune system initially recognizes and eliminates newly transformed cells, the continuous immune pressure selects for tumor cell variants capable of evading immune surveillance. These tumor variants establish an immunosuppressive tumor microenvironment through mechanisms including impaired antigen presentation, recruitment of immunosuppressive cells, secretion of inhibitory cytokines, and upregulation of inhibitory immune checkpoint proteins (ICPs) [4,5].

ICPs are membrane proteins that can be categorized as positive and negative ICPs, which propagate stimulatory or inhibitory signals to immune effector cells, respectively, to maintain immune homeostasis [6]. Under normal conditions, the interactions between inhibitory immune checkpoint molecules on immune effector cells, such as cytotoxic T cells and natural killer (NK) cells, and antigen-presenting cells (APCs) play crucial roles in inhibiting immune effector cell activity to prevent autoimmunity [7]. However, most negative ICPs can be overexpressed in tumor cells as well as the tumor microenvironment, which prevents the cytotoxic response elicited by immune effector cells against tumor cells [8]. Moreover, constant antigen exposure further drives T cell exhaustion, a dysfunctional state characterized by reduced proliferative capacity, cytokine production, cytotoxic function, and sustained expression of ICPs [9]. Tumors can also develop adaptive immune resistance by upregulating inhibitory ligands in response to inflammatory cytokines produced by activated T cells [10]. Collectively, these mechanisms promote immune evasion and tumor progression.

The recognition of these immune evasion mechanisms facilitated the development of immune checkpoint inhibitors (ICIs) to restore antitumor immunity by blocking inhibitory ICPs. In the past 15 years, the United States Food and Drug Administration (FDA) approved several ICIs targeting cytotoxic T-lymphocyte associated protein 4 (CTLA-4), programmed cell death protein-1 (PD-1) and programmed cell death ligand-1 (PD-L1), while various additional ICIs targeting novel ICPs, including T cell immunoglobulin and mucin domain-containing 3 (TIM-3), lymphocyte activation gene-3 (LAG-3), and T cell immunoglobulin and ITIM domain (TIGIT), moved into Phase III clinical trials. Despite these remarkable advances, many patients exhibit primary or acquired resistance to ICIs and treatment-related immune toxicities remain significant clinical concerns. Consequently, considerable efforts have been directed toward developing complementary therapeutic strategies [2].

RNA interference (RNAi) has emerged as a promising strategy for immune checkpoint modulation [11]. By utilizing microRNAs (miRNAs or miRs) and small interfering RNAs (siRNAs), RNAi enables posttranscriptional gene regulation (PTGR) by selectively silencing target messenger RNAs (mRNAs) through degradation or translational repression [12]. MiRNAs exhibit partial complementarity to multiple target transcripts, offering the potential to modulate several immune checkpoint pathways simultaneously [12]. In contrast, siRNAs display perfect complementarity to a single target mRNA, predominantly silencing individual target mRNAs [12]. Preclinical and early clinical studies have demonstrated that different RNAi modalities effectively suppress ICP expression and enhance antitumor immune responses across diverse cancer models, with various delivery platforms facilitating their administration [13,14,15,16]. Despite these encouraging advances, the clinical translation of RNAi-based strategies remains limited by the instability of RNA molecules and inefficient in vivo delivery.

Although immune checkpoint biology and RNA therapeutics have been extensively reviewed separately, this review provides an integrated perspective by linking immune checkpoint biology with RNAi-based therapeutic strategies. We discuss the biological functions of established and emerging ICPs, their ligands, and antibody-based immune checkpoint therapies approved by the FDA or currently under Phase III clinical development. We further summarize the PTGR mechanisms of RNAi and endogenous miRNAs involved in modulating immune checkpoint signaling pathways. We also highlight recent advances in ICP-targeting RNAi-based therapeutics and the delivery platforms used in their preclinical and clinical development. These developments underscore RNAi-based immune checkpoint modulation as a complementary strategy to conventional ICIs in cancer immunotherapy.

## 2. Classical Immune Checkpoint Proteins

### 2.1. CTLA-4

**Figure 1:**
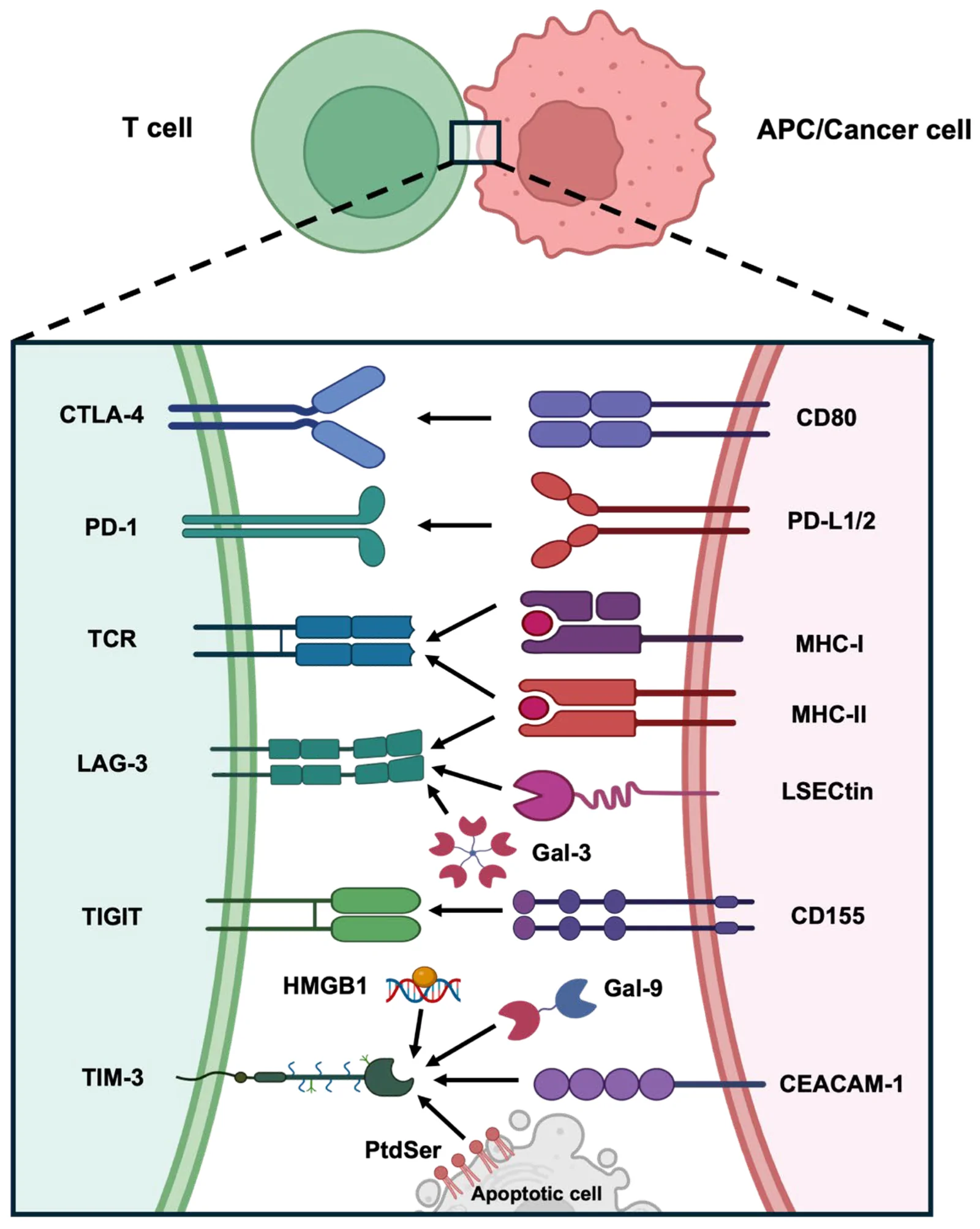
Interactions between ICPs and their ligands critical for T cell activity. Schematic diagram of major immune checkpoint pathways that control T cell activation and inhibition. The TCR is depicted as the primary activating signal counterbalanced by these inhibitory signals. Created in BioRender. Wang, K. (2026) https://BioRender.com/a6zcqdq (accessed on 4 July 2026).

Both CTLA-4 (CD152) and co-stimulatory molecule CD28 belong to the immunoglobulin superfamily and are receptors for B7-1 (CD80) and B7-2 (CD86) ligands expressed on APCs (Figure 1) [17]. Despite their sequence and structural homology, CTLA-4 and CD28 have completely different biological functions [18]. CTLA-4 is mainly expressed on activated and regulatory T lymphocytes (Tregs), acting to dampen T cell activity in both exhausted and regulatory states [19,20]. In contrast, CD28, a co-stimulatory protein essential for T cell receptor (TCR)-mediated T cell activation when interacting with the major histocompatibility complex class I/II (MHC-I/II), is primarily expressed on both naive and activated T cells but is generally absent in exhausted T cells [19]. Both CTLA-4 and CD28 interact with B7 ligands as dimers, but CTLA-4 has higher affinity and avidity to the ligands, compared to CD28 [21,22]. Being mostly an intracellular protein, CTLA-4 cell surface expression is transiently induced on T cells during the early priming phase of CD28-mediated T cell activation in the lymph nodes [23]. Thus, CTLA-4 competes with CD28-B7 interaction, counteracting CD28-mediated co-stimulation of T cells that normally leads to an increase in T cell proliferation, T cell survival, growth, cytokine-mediated T cell differentiation, and energy metabolism [24,25,26]. Interestingly, some reports showed that CTLA-4 is constitutively expressed at different intensities in several human carcinoma, melanoma, neuroblastoma, and sarcoma cell lines [27,28]. Upon treatment with recombinant B7-1 and B7-2 ligands, activation of the CTLA-4 downstream signaling pathway induced caspase-dependent tumor cell apoptosis [27]. In addition, treatment with anti-CTLA-4 antibody induced PD-L1 expression and cell proliferation in NSCLC cell lines through the EGFR pathway, which signifies a potential role of CTLA-4 in cell types besides T cells [29].

Although CTLA-4 expression has been investigated as a prognostic biomarker in cancer, its prognostic value varies widely depending on the tumor type/subtype, the specific cell population expressing CTLA-4 (tumor cells vs. tumor-infiltrating lymphocytes), the disease context (primary tumor vs. metastatic sites), and the immune microenvironment (surrounding CD8^+^ T cell density and the presence of other ICPs) [30,31,32,33]. As CTLA-4 acts mainly in early T cell priming in the lymph nodes and not at the tumor site, its expression often signals immune tolerance rather than local tumor immune evasion.

### 2.2. CTLA-4 Inhibitors Approved by the FDA

Currently, two anti-CTLA-4 antibodies (ipilimumab and tremelimumab) are FDA-approved (Table 1). In 2011, a fully human monoclonal antibody, ipilimumab (brand name: YERVOY^®^, Bristol-Myers Squibb Company, Princeton, NJ, USA), was FDA-approved for the treatment of unresectable or metastatic melanoma [34,35]. In its Phase III trial (NCT00094653), ipilimumab, administered either alone or in combination with a glycoprotein 100 (gp100) peptide vaccine, significantly improved median overall survival (OS) in patients with unresectable stage III or IV melanoma to 10.1 months and 10.0 months, respectively, compared with 6.4 months for gp100 alone. The overall response rates (ORR) were 10.9% for ipilimumab alone and 5.7% for combination with gp100, compared with 1.5% for the gp100 alone group. Responses to ipilimumab were durable, with 60.0% and 17.4% of responders in the monotherapy and combination groups, respectively, maintaining responses for at least 2 years, whereas no responses lasting more than 2 years were observed with gp100 alone [36]. Following the initial approval of ipilimumab monotherapy, ipilimumab plus a PD-1 inhibitor nivolumab combination regimen was later approved for the treatment of melanoma, metastatic NSCLC, advanced renal cell carcinoma, hepatocellular carcinoma (HCC), unresectable malignant pleural mesothelioma (MPM), metastatic esophageal squamous cell carcinoma (ESCC), and metastatic colorectal cancer [35,37,38,39,40,41,42,43,44].

Given the success of ipilimumab in treating advanced melanoma, tremelimumab (brand name: IMJUDO^®^, AstraZeneca Pharmaceuticals LP, Wilmington, DE, USA), another fully human monoclonal antibody specific for CTLA-4, underwent a series of clinical trials for the treatment of melanoma [45]. However, tremelimumab failed to demonstrate a statistically significant improvement in median OS, compared with standard-of-care chemotherapy in patients with metastatic melanoma (12.6 vs. 10.7 months), despite producing significantly more durable responses (35.8 vs. 13.7 months) [46]. Although tremelimumab and ipilimumab both target CTLA-4, the differing clinical outcomes of their melanoma Phase III trials likely reflect a combination of trial design and drug-related factors rather than differences in target engagement alone. The tremelimumab trial was terminated early after an interim futility analysis, while survival follow-up continued, and the final analysis suggested delayed separation of the survival curves [46]. In addition, ipilimumab is an IgG1 antibody, whereas tremelimumab is an IgG2 antibody. The greater affinity of IgG1 for activating Fc*γ* receptors (Fc*γ*R) may promote more effective Fc-dependent depletion of intratumoral Treg cells than IgG2, although clinical evidence has not established Fc-isotype differences as the definitive explanation for the differing efficacy of these antibodies [47,48]. Despite its limited efficacy as monotherapy in advanced melanoma, tremelimumab in combination with the PD-L1 inhibitor durvalumab was first approved in October 2022 for the treatment of unresectable HCC, significantly improving median OS (16.4 vs. 13.8 months), ORR (20.1% vs. 5.1%), and median duration of response (DoR) (22.3 vs. 18.4 months) compared with the sorafenib group [49]. Later in November 2022, tremelimumab combined with durvalumab and platinum-based chemotherapy was also approved for metastatic NSCLC, after demonstrating improvement in median OS to 14.0 months compared with 11.7 months with chemotherapy alone [45,50,51].

### 2.3. PD-1 and PD-L1/2

PD-1 (CD279) is a cell surface protein that is expressed on T cells, B cells, NK cells, macrophages, and some dendritic cells upon antigen recognition and TCR/CD28-mediated activation [52]. PD-1 homodimers interact with its ligands PD-L1 (CD274/B7-H1) and PD-L2 (CD273/B7-DC) dimers, both members of the B7 protein family (Figure 1). Unlike B7-1 and B7-2, which bind to CD28 and CTLA-4, PD-L1 and PD-L2 specifically engage with PD-1. Although they share the same receptor, PD-L1 and PD-L2 differ in their expression patterns, biological functions, and regulatory mechanisms. PD-L1 expression can be readily induced by inflammatory cytokines, especially IFN-γ, on both hematopoietic cells, such as macrophages, dendritic, or B cells, and non-hematopoietic cells, such as epithelial, endothelial, or tumor cells [53,54,55]. PD-L2 expression is more restricted to APCs, especially dendritic cells, and can only be induced by T helper 2 (Th2) cytokines, such as IL-4 or IL-13, on inflammatory macrophages [56,57]. However, this Th2-dependent regulation makes PD-L2 less relevant as a tumor biomarker or cancer therapeutic target in the predominantly T helper 1 (Th1)-oriented tumor microenvironment. The interaction between PD-1 and PD-L1/2 occurs during the later effector phase of T cell activation, where the T cells are located in the peripheral tissues and tumor microenvironment. This interaction can be sustained with chronic antigen stimulation in the case of tumors or persistent infection.

The activation of the PD-1/PD-L1 pathway suppresses TCR signaling, cytokine production, and T cell-mediated cytotoxicity, leading to T cell exhaustion, immune evasion and tumor progression. Consequently, high PD-L1 expression on tumor cells is often associated with poor clinical outcomes, including NSCLC, gastric, breast, colorectal, hepatocellular, urothelial, and renal cancers as well as some hematological malignancies [58,59,60,61,62,63]. While an elevated PD-L1 level in tumors is correlated to poor prognosis, it often predicts a better response to immunotherapy, such as anti-PD-1 and anti-PD-L1 monoclonal antibodies. Most FDA-approved monoclonal antibodies for cancer immunotherapy target PD-1 and PD-L1.

### 2.4. PD-1 Inhibitors Approved by the FDA

Seven ICI medications targeting PD-1 (pembrolizumab, nivolumab, cemiplimab, dostarlimab, retifanlimab, toripalimab, tislelizumab) are approved by the FDA (Table 1), which inhibit the binding of PD-1 to both PD-L1 and PD-L2.

Pembrolizumab (brand name: KEYTRUDA^®^, Merck Sharp & Dohme LLC, Rahway, NJ, USA), an IgG4κ humanized monoclonal antibody, is the first FDA-approved PD-1 inhibitor [64]. In the KEYNOTE-001 Phase Ib study (NCT01295827), patients with unresectable and advanced melanoma following treatment with ipilimumab demonstrated promising ORR of 33% with 44% of responders maintaining their response for over a year and median OS of 23 months, leading to the accelerated approval of pembrolizumab for second-line treatment of advanced or unresectable melanoma in 2014 [65,66,67]. Eventually, the Phase III KEYNOTE-006 trial (NCT01866319) provided definitive evidence that pembrolizumab achieved superior ORR of 33–34%, compared with 12% in the ipilimumab group, leading to full FDA approval of pembrolizumab as a first-line treatment for advanced melanoma [68,69].

Nivolumab (brand name: OPDIVO^®^, Bristol-Myers Squibb Company, Princeton, NJ, USA), a fully human IgG4κ monoclonal antibody, was approved for advanced melanoma as its initial indication in the same year as pembrolizumab, after showing longer median OS (16 vs. 14 months) and higher ORR of (27% vs. 10%) in the chemotherapy arm with fewer treatment-related adverse events in the Phase III CheckMate 037 trial (NCT01721746) [44,70]. Nivolumab is also considered the cornerstone of the dual checkpoint blockade regimen with ipilimumab, targeting two distinct immune checkpoint pathways. In the CheckMate 067 Phase III study (NCT01844505), the combination of nivolumab and ipilimumab resulted in higher ORR (58%) than nivolumab (44%) or ipilimumab (19%) alone. The 2-year OS rate of the combination group was also higher (64%) than either nivolumab (59%) or ipilimumab (45%) alone in patients with advanced melanoma [71]. Importantly, long-term follow-up from this trial demonstrated that patients who discontinued combination therapy due to treatment-related toxicities often maintained durable clinical benefit and OS, suggesting that effective antitumor immunity may persist after treatment discontinuation [71].

Cemiplimab (brand name: LIBTAYO^®^, Regeneron Pharmaceuticals, Inc., Tarrytown, NY, USA) is a fully human IgG4 monoclonal antibody and is the third FDA-approved anti-PD-1 therapy. Approved in 2018, cemiplimab is the first medication for patients with metastatic or locally advanced cutaneous squamous cell carcinoma (cSCC) who are not candidates for curative surgery or radiation [72,73]. The approval was supported by an ORR of 47%, with 57% of responders maintaining a DoR of more than 6 months in the Phase II study (NCT02760498) of cemiplimab [74]. In 2021, cemipllimab was also FDA-approved as the first immunotherapy for the treatment of advanced basal cell carcinoma (BCC), demonstrating an ORR of 21.4% with all responders maintaining their response for at least 8 months [75]. Given that BCC and cSCC remain the most and second most common skin cancers, respectively, the FDA approval of cemiplimab represents a significant advancement in cancer immunotherapy. More recently, cemiplimab alone or in combination with chemotherapy was approved by the FDA as first-line treatment for advanced NSCLC with at least 50% tumor cells expressing PD-L1 [73,76,77]. Cemiplimab monotherapy demonstrated superior clinical outcomes over chemotherapy, with an estimated 24-month OS rate of 50.0% vs. 27.0% and median DoR of 16.7 vs. 6.0 months [76]. In combination with chemotherapy, cemiplimab also significantly improved survival compared with placebo plus chemotherapy, achieving a median OS of 21.9 vs. 13.0 months and extending the median DoR to 15.6 vs. 7.3 months [77].

In 2021, the FDA approved a humanized IgG4 monoclonal antibody called dostarlimab (brand name: JEMPERLI^®^, GlaxoSmithKline LLC, Philadelphia, PA, USA) initially indicated to treat patients with mismatch repair deficient (dMMR) recurrent or advanced endometrial cancer [78,79]. Dostarlimab was then approved as a single agent for the treatment of recurrent or advanced solid tumors that are dMMR and in combination with chemotherapy for the treatment of dMMR endometrial cancer followed by dostarlimab monotherapy [79,80,81]. In the GARNET trial (NCT02715284) evaluating the antitumor activity of patients with dMMR endometrial cancer, dostarlimab monotherapy demonstrated an ORR of 42.3%, and the median OS was not reached, indicating durable clinical benefit [82]. In the RUBY trial (NCT03981796), dostarlimab in combination with carboplatin and paclitaxel significantly improved the 24-month OS rate to 71.3% compared with 56.0% for placebo plus chemotherapy in patients with dMMR recurrent or advanced endometrial cancer [81].

Retifanlimab (brand name: ZYNYZ^®^, Incyte Corporation, Wilmington, DE, USA) is a humanized IgG4 monoclonal antibody that emerged as a significant advancement in cancer immunotherapy following its 2023 FDA approval [83]. It is initially indicated for the treatment of metastatic or recurrent locally advanced Merkel cell carcinoma (MCC), a rare and highly aggressive skin cancer. This accelerated approval was supported by findings from the POD1UM-201 (NCT03599713) trial, which demonstrated a 52.3% ORR with 62.0% of responders maintaining a DoR of at least 12 months in patients with advanced MCC [84]. Another clinical breakthrough of retifanlimab lies in the management of squamous cell carcinoma of the anal canal (SCAC). As established by the POD1UM-202 (NCT03597295) study, retifanlimab as a monotherapy has been approved as a second-line therapy for patients with SCAC, showing an ORR of 13.8%, a median DoR of 9.5 months, and a median OS of 10.1 months [85]. Similar antitumor activity and safety profiles were seen in the POD1UM-303 (NCT04472429) trial, where the integration of retifanlimab into first-line carboplatin/paclitaxel chemotherapy significantly improved clinical outcomes in patients with locally recurrent or metastatic SCAC, with a median OS advantage of 29.2 vs. 23.0 months for placebo plus chemotherapy, an ORR of 55.8% vs. 44.2%, and a median DoR of 14.0 vs. 7.2 months, ultimately leading to its FDA-approval in 2025 [86].

In 2023, the FDA approved toripalimab (brand name: LOQTORZI^®^, Coherus BioSciences, Inc., Redwood City, CA, USA), a humanized IgG4 monoclonal antibody, in combination with chemotherapy and as monotherapy as the first-line treatment of adult patients with metastatic or recurrent nasopharyngeal carcinoma (NPC) that has progressed during or after standard chemotherapy [87]. The approval of toripalimab as monotherapy was supported by the POLARIS-02 Phase II trial (NCT02915432), which demonstrated an ORR of 20.5% with a median DoR of 12.8 months, and a median OS of 17.4 months [88]. The approval of toripalimab in combination with gemcitabine and cisplatin was supported by the JUPITER-02 Phase III trial (NCT03581786), where the 3-year OS rate was 64.5% vs. 49.2% in the placebo plus chemotherapy group and ORR of 78.8% vs. 67.1%, respectively [89]. Since chemotherapy was the standard first-line treatment for patients with metastatic or recurrent NPC, toripalimab’s approval as the first immunotherapy for NPC set a new standard of care for the first-line treatment of metastatic or recurrent NPC, significantly impacting clinical practice.

Tislelizumab (brand name: TEVIMBRA^®^, BeOne Medicines USA, Inc., Pennington, NJ, USA) is a humanized IgG4 monoclonal PD-1 blocking antibody engineered to minimize binding to Fc*γ*R on macrophages, thereby reducing Fc*γ*R-mediated crosslinking activity compared with traditional anti-PD-1 antibodies, such as pembrolizumab and nivolumab [90,91]. Tislelizumab was first approved in 2024 as second-line treatment in patients with advanced ESCC, based on the results from the Phase III RATIONALE-302 (NCT03430843), in which tislelizumab significantly improved median OS (8.6 vs. 6.3 months), ORR (20.3% vs. 9.8%), and median DoR (7.1 vs. 4.0 months) compared with chemotherapy (paclitaxel, docetaxel, or irinotecan) [92]. The Phase III RATIONALE-306 (NCT03783442) study demonstrated that the addition of tislelizumab to platinum plus fluoropyrimidine or paclitaxel significantly improved median OS (17.2 vs. 10.6 months) and ORR (63.0% vs. 42.0%), compared with placebo plus chemotherapy, supporting its FDA approval as a first-line treatment for advanced ESCC in combination with chemotherapy [93].

### 2.5. PD-L1 Inhibitors Approved by the FDA

Three PD-L1 inhibitors (atezolizumab, avelumab, and durvalumab) are currently approved by the FDA (Table 1). These antibodies only disrupt the binding of PD-L1 to PD-1, while keeping the PD-1/PD-L2 interaction intact. This strategy helps maintain peripheral immune tolerance, minimizing the risk of generalized inflammation and autoimmune toxicities [94].

Atezolizumab (brand name: TECENTRIQ^®^, Genentech, Inc., South San Francisco, CA, USA) is a humanized IgG1 monoclonal PD-L1 blocking antibody, which was the first anti-PD-L1 antibody approved by the FDA in 2016, indicated for urothelial carcinoma as a second-line therapy, based on the results of the Phase II IMvigor210 (NCT02108652) study [95,96,97]. In this study, patients were stratified according to the percentage of PD-L1 expression on tumor-infiltrating cells (ICs), defined as IC0 (<1%), IC1(≥1% to <5%), and IC2/3 (≥5%). In the primary analysis, atezolizumab significantly improved the ORR to 27.0% in the IC2/3 group and 18% in the IC1/2/3 group, compared with the historical control rate of 10.0%. Median OS was 11.4 months in the IC2/3 group and 8.8 months in the IC1/2/3 group. The median DoR was not reached, indicating durable clinical benefit [96]. Atezolizumab also expanded immunotherapy into first-line combination regimens for metastatic NSCLC in the Impower150 (NCT02366143) trial and HCC in the IMbrave150 (NCT03434379) study [98,99].

Avelumab (brand name: BAVENCIO^®^, EMD Serono, Inc., Boston, MA, USA) is a human IgG1 lambda monoclonal PD-L1 antibody initially approved in 2017 for the treatment of metastatic MCC [100,101]. Avelumab is unique among PD-L1 inhibitors as it preserves Fc-mediated effector function, potentially enabling engagement of NK cells to induce antibody-dependent cellular cytotoxicity in addition to PD-L1 blockade, a feature absent in other clinically approved agents in this class [102,103]. While this Fc region allows for a potential dual mechanism of action, its toxicity profile remains highly manageable and comparable to other checkpoint inhibitors [104,105]. The Phase II JAVELIN Merkel 200 (NCT02155647) trial demonstrated durable survival outcomes in patients with metastatic MCC, further establishing avelumab as a standard-of-care treatment option for this aggressive malignancy [106]. After a minimum of 44 months follow-up, the median OS was 12.6 months, ORR was 33%, and median DoR was 40.5 months [106].

Durvalumab (brand name: IMFINZI^®^, AstraZeneca Pharmaceuticals LP, Wilmington, DE, USA) is a fully human immunoglobulin IgG1*κ* monoclonal anti-PD-L1 antibody that was first approved by the FDA in 2017 for the treatment of locally advanced or metastatic urothelial carcinoma previously treated with platinum-containing chemotherapy [107,108]. In the pivotal trial (NCT01693562), durvalumab achieved an ORR of 17.8%, with higher responses observed in patients with high PD-L1 expression (27.6%) than in patients with low/negative PD-L1 expression (5.1%). The median OS was 18.2 months in the overall patient population, with an increase to 20.0 months in the high PD-L1 population, compared with 8.1 months in the low/negative PD-L1 population. The median DoR was not reached [109]. Durvalumab gained much broader recognition following the landmark PACIFIC (NCT02125461) trial, in which durvalumab demonstrated significantly higher estimated 5-year OS rate (42.9% vs. 33.4%), ORR (29.8% vs. 18.3%), and median DoR (not reached vs. 18.4 months), compared with placebo [110]. These results led to its approval in 2018 as consolidation therapy for unresectable stage III NSCLC after concurrent chemoradiotherapy [107,111]. This indication transformed the therapeutic landscape and contributed substantially to the widespread adoption of duvalumab in cancer therapy.

## 3. Novel ICI in Phase III Clinical Trials

### 3.1. TIM-3

First discovered in 2002, TIM-3 is a transmembrane protein primarily expressed on IFN-γ-producing CD4^+^ Th1 cells and CD8^+^ cytotoxic T cells, where it functions as a negative regulator of type 1 immune responses [112]. In cancer, TIM-3 is frequently upregulated on exhausted T cells, Tregs, monocytes, dendritic cells, NK cells, and other immune populations within the tumor microenvironment [112,113,114,115,116]. TIM-3 exerts its immunosuppressive effects through interactions with multiple ligands, including galectin-9 (Gal-9), high-mobility group protein B1 (HMGB1), a non-protein molecule phosphatidylserine (PtdSer), and carcinoembryonic antigen cell adhesion molecule-1 (CEACAM-1) in various cellular contexts (Figure 1) [117,118,119,120]. Among these, the TIM-3/Gal-9 pathway is the most extensively characterized. Gal-9 binding induces intracellular calcium influx and apoptosis in Th1 cells, suppresses IFN-γ production, and promotes T cell exhaustion, which weakens antitumor immunity [117]. HMGB1, an alarmin released by stressed or dying cells, normally facilitates dendritic cell recognition of tumor-derived nucleic acids and promotes innate immune activation. However, TIM-3 binding prevents HMGB1 from transporting nucleic acids into endosomes, impairing innate immune sensing and reducing antitumor immune activation [119]. TIM-3 also recognizes PtdSer, a phospholipid exposed on apoptotic cells, contributing to apoptotic cell clearance and maintenance of immune tolerance [118,121]. Excessive interaction between TIM-3 and PtdSer in the tumor microenvironment may promote tolerogenic immune responses. CEACAM-1 functions both as a TIM-3 ligand and a regulatory partner that promotes TIM-3 maturation and inhibitory signaling, facilitating T cell exhaustion and immune tolerance [120]. High TIM-3 expression is detected in various cancer tissues, including NSCLC, gastric cancer, renal cell carcinoma, bladder urothelial carcinoma, colon cancer, and HCC [122,123,124,125,126,127]. Thus, the abovementioned ligand-dependent mechanisms establish TIM-3 as a central mediator of immune suppression in cancer and a promising target for cancer immunotherapy.

Several anti-TIM-3 antibodies have entered clinical trials for the treatment of advanced malignancies. Among the candidates, sabatolimab (MBG453), a humanized IgG4 monoclonal antibody, is one of the first anti-TIM-3 antibodies to reach Phase III clinical trials. Preclinical data demonstrated that sabatolimab enhances T cell cytotoxic effects and myeloid cell inflammatory cytokine production, promotes antibody-mediated cell phagocytosis of TIM-3^+^ leukemic stem cells, and disrupts the interaction between TIM-3 and its ligands (Gal-9 and PtdSer) by limiting accessibility to the binding site [128]. Early clinical studies combining sabatolimab with hypomethylating agents in higher-risk myelodysplastic syndromes and acute myeloid leukemia (AML) showed encouraging efficacy and tolerability, leading to the initiation of several larger trials [129]. However, the pivotal Phase III STIMULUS-MDS2 (NCT04266301) trial failed to demonstrate a significant OS benefit, leading to the discontinuation of the late-stage development [130]. Nevertheless, sabatolimab remains the most clinically advanced anti-TIM-3 antibody to date and has provided important evidence supporting TIM-3 as a therapeutic target in cancer immunotherapy.

### 3.2. LAG-3

LAG-3 was identified in 1990 as a molecule highly expressed on activated effector CD4^+^ T cells, CD8^+^ T cells, and NK cells [131]. LAG-3, sharing high structural similarity with the CD4 receptor, interacts with MHC-II molecules on APCs with a greater affinity than CD4, which inhibits CD4^+^ T cell function (Figure 1) [132]. LAG-3 also interacts with liver and lymph node sinusoidal endothelial cell C-type lectin (LSECtin) that functions as a non-canonical ligand extending LAG-3-mediated inhibition beyond CD4^+^ T cells to CD8^+^ T cells and NK cells (Figure 1) [133,134]. LSECtin has been proposed to bind to N-linked glycans on the extracellular domain of LAG-3. Because LSECTin recognizes carbohydrate moieties rather than specific peptide sequences, the interaction is believed to be mediated by the glycosylation status of LAG-3 [135]. Binding of LSECtin to glycosylated LAG-3 suppresses effector T-cell function and reduces IFN-γ production [133,136]. Similarly, galectin-3 (Gal-3), a β-galactoside-binding lectin secreted by numerous immune and tumor cells, binds to glycosylated regions of LAG-3 on activated T cells and cross-links receptors, promoting LAG-3 clustering and strengthening inhibitory signaling (Figure 1) [137,138].

LAG-3 is frequently co-expressed with PD-1 on tumor-infiltrating exhausted T cells across multiple malignancies, such as NSCLC, HCC, and colorectal cancer [139,140,141]. These inhibitory receptors act in a redundant and compensatory manner to maintain T cell dysfunction, limiting the efficacy of LAG-3 blockade as a single agent. Therefore, LAG-3 blockade therapies now undergoing clinical trials are mostly bispecific antibodies targeting both LAG-3 and PD-1/PD-L1 or anti-LAG-3 antibodies used in combination with PD-1/PD-L1 blocking antibodies [142].

Relatlimab is a first-in-class monoclonal antibody targeting LAG-3, marking a major milestone in cancer treatment (Table 1). It was first approved by the FDA in 2022 only in combination with PD-1 inhibitor nivolumab as a single fixed-dose vial (brand name: OPDUALAG^®^, Bristol-Myers Squibb Company, Princeton, NJ, USA) for the treatment of unresectable or metastatic melanoma [143,144]. This approval was based on the RELATIVITY-047 trial (NCT03470922), which demonstrated improved progression-free survival (PFS) with OPDUALAG^®^ compared with nivolumab monotherapy (10.1 vs. 4.6 months), reducing the risk of disease progression or death by 25% [145].

### 3.3. TIGIT

TIGIT, mainly expressed on activated, regulatory, memory T cells and NK cells, was discovered in 2009 through an Immune Response In Silico microarray as a novel inhibitory receptor containing protein structures that resemble immunomodulatory receptors [146,147]. TIGIT, a member of the CD28 protein family, interacts with both CD155 (PVR) and CD112 (PVRL2, nectin-2) on APCs and tumor cells with a higher binding affinity to CD155, which delivers a co-inhibitory signal to immune cell function (Figure 1) [147,148,149]. The co-stimulatory receptor CD226 (DNAM-1) on T cells and NK cells also binds to CD155, but TIGIT exhibits a higher binding affinity for CD155, competing with CD226. TIGIT can also disrupt the homodimerization of CD226 through direct binding, inhibiting its co-stimulatory function. However, more studies are needed to elucidate the co-expression and dynamics of TIGIT and CD226 on T and NK cells in the tumor microenvironment or inflamed tissues, which is likely context-dependent. The functional significance of the interaction between TIGIT and CD112 is not fully understood.

Since TIGIT and PD-1 are often co-expressed on tumor-infiltrating lymphocytes, treatment with anti-TIGIT monotherapy failed to fully activate immune cells for antitumor efficacy in multiple pre-clinical tumor models, while blocking TIGIT and PD-1/PD-L1 antibodies has shown elevated antitumor activity [150,151,152,153]. Therefore, similar to anti-LAG3 therapies, anti-TIGIT is often administered in combination with other immunotherapy agents, especially anti-PD-1/PD-L1 agents. Currently, numerous anti-TIGIT antibodies have entered clinical evaluation, primarily in combination with other anti-cancer agents. Among these, four have reached Phase III trials, including tiragolumab, vibostolimab, domvanalimab, ociperlimab, yet none has received FDA approval. In early-phase trials, combinations of these anti-TIGIT agents with PD-1 or PD-L1 inhibitors initially showed encouraging response rates and immune activation, with tiragolumab being the first anti-TIGIT agent granted breakthrough therapy designation by the FDA. However, most programs failed to demonstrate meaningful improvements in OS compared with established standard PD-1 or PD-L1 therapy alone, while increasing immune-related toxicities, leading to the termination of multiple Phase III studies [154,155,156,157,158,159]. The repeated failed and discontinued trials across tiragolumab, vibostolimab, domvanalimab, ociperlimab suggest that TIGIT likely functions as a context-dependent rather than dominant immune checkpoint that is insufficient to generate durable survival improvement in a broader patient population, highlighting the need for a more complete mechanistic understanding of TIGIT biology and better predictive biomarkers to identify patients most likely to benefit from TIGIT blockade.

## 4. PTGR via miRNAs and siRNAs

ICI using monoclonal antibodies has established a foundational framework for clinical cancer immunotherapy, demonstrating that modulation of immune inhibitory pathways can produce durable antitumor responses in subsets of patients. This success has also catalyzed interest in expanding the range of therapeutic modalities capable of fine-tuning immune regulatory networks. RNAi-based therapies, exploiting the mechanisms of miRNA and siRNA, have emerged as a complementary and mechanistically distinct approach.

MiRNAs are genome-derived, single-stranded, small non-coding RNAs that are typically 21–22 nucleotides (nt) in length. MiRNAs primarily regulate their target gene expression on the mRNA level through PTGR, i.e., mRNA degradation or translational inhibition [160]. Lin-4 was the first miRNA discovered with the ability to regulate lin-14 during the developmental process in *Caenorhabditis elegans* [161], followed by the identification of let-7, which is the first known human miRNA [162]. The presence of miRNAs has been well-documented and highly conserved across diverse animal species, from invertebrates to mammals [163,164,165]. MiRNA-mediated PTGR plays important roles in various cellular processes, such as development, cell differentiation, and metabolism [166,167]. Disruptions in miRNA function are involved in various human diseases and malignancies [168].

**Figure 2:**
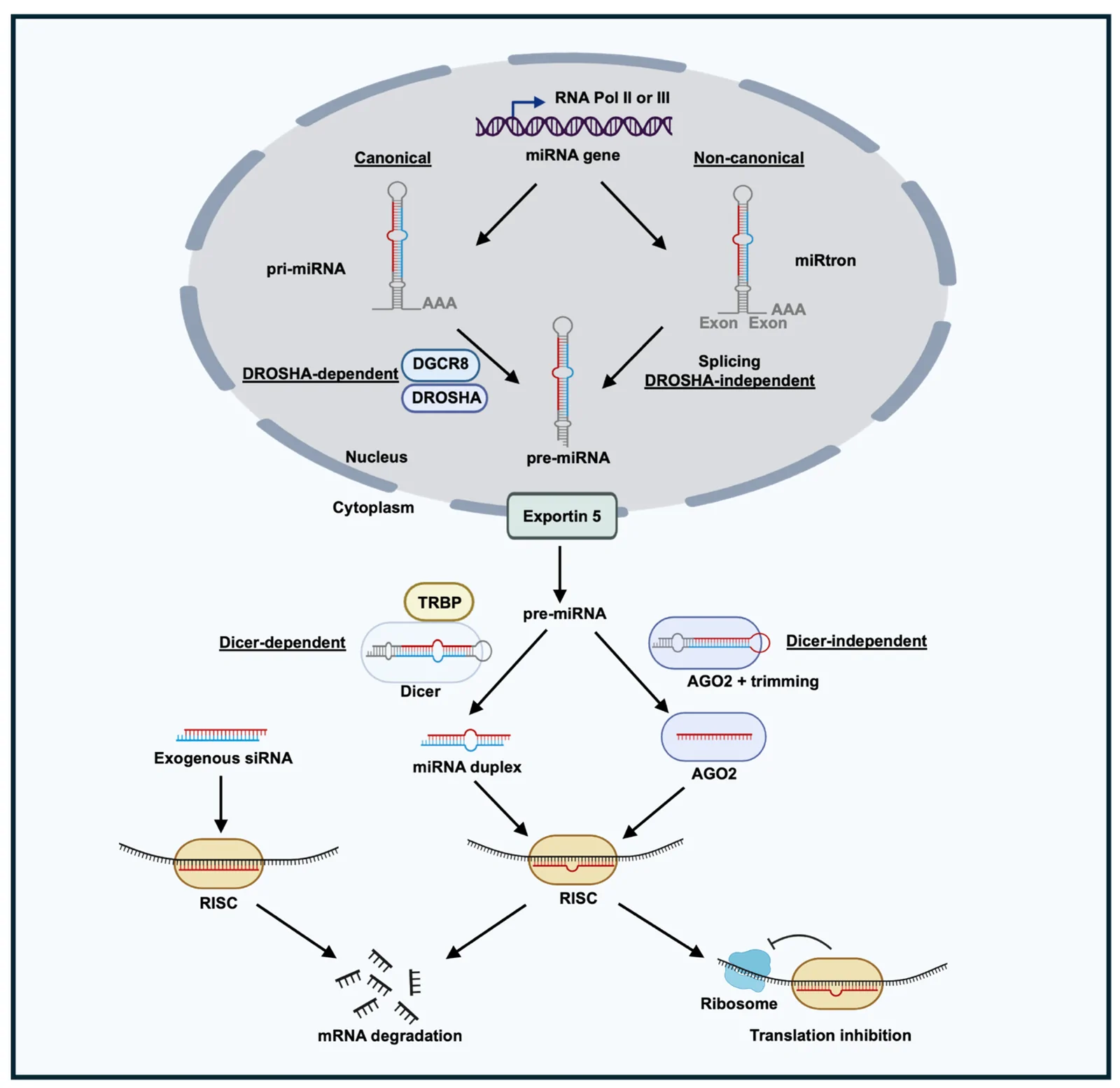
RNAi mechanisms mediated by miRNA and siRNA in mammalian cells. MiRNAs originate from pri-miRNAs, which are processed by DROSHA- dependent or -independent pathways into pre-miRNAs in the nucleus. After being exported into the cytoplasm via exportin 5, pre-miRNAs are cleaved by Dicer-dependent or -independent pathways. The guide strands (red) are preferably incorporated into RISC, typically interacting with the 3′UTR of target mRNAs through imperfect complementarity and resulting in translational repression or mRNA degradation, while the passenger strands (blue) are degraded. Double-stranded siRNAs are exogenously introduced into the cytoplasm, and the antisense strands (red) are loaded into RISC to guide cleavage of target mRNAs at the CDS or 3′UTR through (near)-perfect complementarity, leading to mRNA cleavage and degradation. Together, miRNAs and siRNAs converge into the RNAi pathway to exert PTGR. Created in BioRender. Wang, K. (2026) https://BioRender.com/eqanfur (accessed on 4 July 2026).

MiRNA biogenesis is a complex and multistep process to ensure proper function of the mature miRNA, consisting of canonical and noncanonical pathways (Figure 2). In the canonical pathway, miRNA genes in the genome are transcribed by RNA polymerase II or III into primary miRNAs (pri-miRNAs), which are more than 1000 nt long and contain RNA stem-loop structures. The pri-miRNA is then processed in the nucleus by the microprocessor complex, consisting of a ribonuclease III enzyme called DROSHA and a dsRNA-binding protein called DiGeorge critical region 8 (DGCR8), at 11 base pairs from the base of the hairpin stem into an approximately 70 nt long precursor miRNA (pre-miRNA) containing a 3′ overhang [169]. By forming a complex with the exportin-5 transporter and the small nuclear GTPase RanGTP, the pre-miRNA is exported from the nucleus into the cytoplasm mediated by GTP hydrolysis [170,171]. In the cytoplasm, pre-miRNA is then further cleaved by another ribonuclease III enzyme, Dicer, at the termini of the stem-loop structure, in complex with the transactivating response RNA-binding protein (TRBP), into a 22 nt long miRNA duplex [172]. Argonaute 1-4 (AGO1-4) endonuclease, recruited by the Dicer/TRPB complex [173], unwinds the miRNA duplex [174], and preferentially selects either the 5p or 3p strand exhibiting a lower internal stability as the guide strand [175] that is loaded into the RNA-induced silencing complex (RISC) [176], while the passenger strand is degraded [177]. The mature miRNA within the miRNA-RISC complex interacts with its target mRNA sequence through partial complementary base-pairing to the 3′ untranslated region (3′UTR), allowing for a wide array of mRNA targets. However, the seed sequence located at positions 2–7 from the guide strand 5′ end is perfectly complementary to multiple sites on its target mRNAs called miRNA response elements (MREs) for effective RNAi [178].

Besides the canonical pathway, non-canonical miRNA biogenesis pathways are categorized into DROSHA/DGCR8- and Dicer/TRBP-independent pathways (Figure 2). Debranched introns on mRNAs with structures resembling pri-miRNAs called miRtrons undergo miRNA processing through splicing that is independent of DROSHA/DGCR8. The spliced product enters the Dicer/TRBP-dependent pathway due to its structural similarity to Dicer substrates [179]. On the contrary, while still processed by DROSHA/DGCR8, miR-451-5p is a well-established miRNA that undergoes a fully Dicer/TRBP-independent maturation pathway, which involves direct cleavage by AGO2 on the 3p arm of pre-miR-451 [180,181,182]. This intermediate product is further trimmed at the 3′ end by the poly(A)-specific ribonuclease (PARN) to produce mature 5p miRNA for RNAi [182,183].

In contrast, therapeutic siRNAs are exogenous, chemically synthesized double-stranded RNA molecules approximately 21–22 nt in length that are introduced directly into the cytoplasm, bypassing nuclear processing. Unlike endogenous miRNAs that require sequential processing by DROSHA/DGCR8 and Dicer/TRBP, therapeutic siRNAs are already antisense–sense duplexes and are unwound in the cytoplasm, where the antisense (guide) strand is preferentially incorporated into the RISC, while the sense (passenger) strand is degraded. Although miRNAs can be functionally associated with AGO1-4 proteins, AGO2 is the only human AGO protein possessing endonucleolytic activity and serves as the primary effector of siRNA-mediated gene silencing [175,184]. The siRNA antisense strand is designed to exhibit perfect or near-perfect complementarity to the protein-coding sequence (CDS) or 3′UTR of one target mRNA, which directs AGO2-mediated endonucleolytic cleavage of the transcript, leading to rapid degradation of the cleaved products and gene silencing [184,185,186]. Therapeutically, synthetic miRNA mimics are designed to restore the function of endogenous miRNAs. While both therapeutic miRNA mimics and siRNAs bypass endogenous miRNA biogenesis and are delivered directly into the cytoplasm, miRNA mimics exploit the endogenous miRNA mechanism by recognizing multiple target transcripts through partial sequence complementarity, resulting in translational repression through steric blockage and/or mRNA degradation through the recruitment of mRNA decay machinery rather than AGO2-mediated cleavage [12,187,188].

## 5. MiRNA-Mediated PTGR of ICPs

Given the mechanisms of PTGR, miRNAs have emerged as critical posttranscriptional regulators of ICPs and their ligands, playing critical roles in shaping antitumor immune responses across various cancer types. Growing evidence demonstrated that specific miRNAs negatively modulate major inhibitory pathways, such as CTLA-4, PD-1, PD-L1, and other checkpoint-associated molecules, by directly binding to the 3′UTR of targeted mRNAs (Table 2).

Several miRNAs have been identified to downregulate CTLA-4 expression in T cell subsets, particularly in Tregs, which are important mediators of immune tolerance. Research on CD4^+^ Tregs has historically dominated the literature due to their well-established role in maintaining immune homeostasis [227,228]. miR-145-5p and miR-138-5p have been reported to directly reduce CTLA-4 expression levels in healthy human CD4^+^ peripheral blood Tregs, where miR-138-5p treatment demonstrated immune-mediated anti-glioma efficacy in mouse models [190,191]. In contrast, CD8^+^ Tregs remain underexplored but are increasingly recognized for their significant contributions in immunoregulatory roles [227,228,229]. Emerging evidence indicates that miR-155-5p and miR-9-5p can directly downregulate CTLA-4 levels in healthy human CD8^+^ umbilical cord blood Tregs [189]. As both CD4^+^ and CD8^+^ Tregs become dysregulated and elevated in cancer, these studies emphasize the promise of using miRNAs to target ICPs expressed on Tregs in cancer [230].

Similarly, PD-1 expression is subject to miRNA-mediated control. Due to the species-conserved MRE site of miR-28-5p on human and mouse PD-1 mRNA, Li et al. [192] demonstrated that miR-28-5p inhibited PD-1 levels and recovered the release of cytokines, such as IL-2 and TNF-α, in exhausted Tregs of melanoma-bearing mice. MiR-374b-5p suppresses PD-1 mRNA levels in healthy human cytokine-induced killer cells [193]. Notably, miR-138-5p targets both CTLA-4 and PD-1 proteins in glioma models [191], indicating a dual regulatory role that amplifies its immunomodulatory effects.

PD-L1 is the most extensively regulated ICP ligand by miRNAs across multiple malignancies. In gastrointestinal cancers, miR-105-5p, miR-502-5p, and miR-152-3p decrease PD-L1 both mRNA and protein levels in gastric cancer [194,196,210], while miR-148a-3p, miR-138-5p, and miR-93-5p exert similar effects in colorectal cancer [209,214,217].

In thoracic and related cancers, miR-200a/b/c-5p, miR-34a-5p, miR-140-5p, miR-138-5p, and let-7-5p family members have been shown to suppress PD-L1 expression in NSCLC, contributing to inhibition of tumor cell growth, metastasis, and enhanced antitumor immunity [202,204,205,212,215]. Additionally, tumor suppressor miR-193a-3p and miR-15a/15b/16-5p cluster downregulated PD-L1 mRNA and protein in MPM [197].

Triple-negative breast cancer (TNBC) cells have been reported to express significantly elevated levels of PD-L1 compared with other breast cancer subtypes [199,231]. Particularly, miR-195-5p, miR-497-5p, and miR-93-5p directly downregulate PD-L1 expression in TNBC, highlighting their potential in immunotherapeutic strategies in TNBC [199,218]. One study further demonstrated that let-7-5p family members can downregulate PD-L1 expression in MCF-7 cells, a non-TNBC breast cancer model expressing estrogen receptor and progesterone receptors [212]. This finding suggests that miRNA-mediated regulation of PD-L1 is not restricted to TNBC but may extend across multiple breast cancer subtypes. Similarly, in hematological malignancies such as AML and diffuse large B-cell lymphoma, miR-200-5p, miR-34a-5p, let-7-5p and miR-195-5p suppress PD-L1 [198,201,212]. Furthermore, miR-497-5p extends its regulatory role in renal cell carcinoma [200]. These findings reinforce the role of these miRNAs as conserved regulators across multiple solid and liquid tumors.

In gynecological tumors, miR-140-5p, miR-142-5p, miR-340-5p, miR-383-5p, and let-7-5p target PD-L1 in cervical cancer, while miR-424-5p regulates PD-L1 in ovarian cancer in the context of chemoresistance [207,212,216]. In other solid tumors, PD-L1 expression is suppressed by miR-145-5p in bladder cancer, miR-140-5p and let-7-5p in osteosarcoma, miR-142-5p in pancreatic cancer, miR-17-5p in melanoma, and miR-217-5p in laryngeal cancer [195,206,208,211,212,213].

Beyond the classical CTLA-4 and PD-1/PD-L1 axes, miRNAs also target novel checkpoint molecules and their associated molecules. miR-379-5p regulates TIM-3 and TIGIT expression in CD8^+^ T cells in breast cancer models [219]. Gal-9, the primary ligand for TIM-3, is targeted by miR-445-5p in skull base chordoma and colon cancer and by miR-22-3p in liver cancer [220,221,222]. CEACAM-1, another ligand for TIM-3, is suppressed by miR-423-5p in NSCLC [223]. HMGB1, an additional ligand for TIM-3, is targeted by miR-1284-5p in osteosarcoma, miR-142-5p in cervical cancer, and miR-505-3p in glioma [224,225,226].

## 6. ICP-Targeting RNAi Drugs Under Development

Several RNAi-based therapeutics targeting ICPs are currently under preclinical and early clinical development, highlighting the growing interest in RNAi strategies for cancer immunotherapy. Current investigations predominantly focus on the inhibition of CTLA-4, PD-1, PD-L1, and TIGIT using siRNA platforms (Table 3).

As intracellular delivery remains one of the major challenges limiting the clinical translation of RNA therapeutics, the incorporation of advanced delivery systems or modifications to improve delivery efficiency and tumor specificity is essential. Naked RNA exhibits poor metabolic stability and pharmacokinetic profiles because it is susceptible to endogenous nuclease degradation, rapid renal clearance due to its small molecular size relative to the kidney filtration threshold, and poor cellular uptake due to its strong negative charge and relatively large size to diffuse across cell membranes [250,251,252]. To overcome these barriers, a broad range of delivery strategies, including polymeric/lipid-based nanoparticles, inorganic nanocarriers, biomimetic vesicles, ligand-mediated delivery systems, and carrier-free RNA platforms, have been developed to enhance RNA stability, tumor-specific accumulation, cellular internalization, and intracellular release (Table 3).

Among these delivery strategies, polymeric/lipid-based nanocarriers have been widely explored for siRNA targeting CTLA-4. Cationic lipid-assisted poly (ethylene glycol)-*block*-poly (_D,L_-lactide) (PEG-PLA)-based nanoparticles have demonstrated promising outcomes for CTLA-4 siRNA delivery in melanoma [13]. Similarly, dual targeting of CTLA-4 and PD-1 has been achieved using poly (_D,L_-lactide-*co*-glycolide) (PLGA)-PEG-based cationic lipid–polymer nanoparticles carrying both CTLA-4 aptamer and PD-1 siRNA in melanoma and colon cancer models [236]. In contrast, by incorporating hydrophobic moieties along with chemical modifications, the INTASYL™ self-delivering siRNA platform eliminates the need for an external carrier, while maintaining efficient intracellular delivery, and has also shown encouraging preclinical efficacy with or without PD-1 combination therapy [13,232,233,234]. Besides nanoparticle-mediated delivery, CTLA-4 itself has also been exploited as a means for delivery in addition to a therapeutic target, because it undergoes constitutive and rapid internalization from the cell surface. Based on this strategy, a CTLA-4 aptamer fused to siSTAT3 has shown encouraging STAT3 silencing and tumor suppressive activity in melanoma, renal cell carcinoma, lymphoma, and colon carcinoma models, following the internalization of CTLA-4 on tumor-associated CD4^+^ and CD8^+^ T cells or malignant T cells [235].

PD-1 and PD-L1 remain the most extensively investigated ICP targets for RNAi-based immunotherapy. Notably, the PD-1-targeting INTASYL™ self-delivering siRNA candidate PH-762 has advanced into Phase I clinical (NCT06014086) evaluation for cSCC, melanoma, and Merkel cell carcinoma, representing one of the most clinically advanced ICP-targeting RNAi therapeutics [14,237]. Interim results from the ongoing clinical trial of PH-762 have demonstrated favorable tolerability without apparent systemic or off-target toxicities, together with encouraging tumor response [253].

Most PD-L1-directed RNAi agents remain at the preclinical stage but utilize a diverse array of nanotechnology-based delivery systems. Polymeric/lipid-based systems, including acidity-responsive micelleplexes combined with photodynamic therapy and polycaprolactone-polyethylene glycol and polycarprolactone-polyethylenimine (PCL-PEG/PCL-PEI)-based hybrid micelles, have been developed to improve intracellular delivery and siRNA stability in melanoma models [238,239,241]. A peptide-branched polymer conjugate with lipid/siRNA complex, a hybrid delivery system, consists of a peptide-branched polymer that provides targeting to tumors and facilitates cellular uptake and endosomal escape, while the lipid/siRNA complex protects the siRNA and elicits PD-L1 silencing in NSCLC models [243]. Inorganic nanocarriers, including gold-CGKRK peptide (Au-CGKRK) nanoconjugates co-delivering PD-L1 and STAT3 siRNA, iron oxide-based magnetic nanoparticles, and PEGylated PEI-coated bismuthene nanoparticles have been investigated to enhance tumor-directed delivery of PD-L1 siRNA and intracellular uptake while simultaneously providing therapeutic functionalities, such as peptide-mediated targeting, magnetic responsiveness, or photothermal therapy, in melanoma, pancreatic cancer, and breast cancer models [240,242,244]. Biomimetic carriers, such as macrophage membrane-camouflaged polycationic nanoparticles, have been developed to enhance tumor-specific delivery through tumor-associated biomarkers, intracellular uptake, and siRNA stability in breast cancer models [245].

Additionally, a biological small interfering RNA (BioRNA/siRNA) agent against PD-L1 developed through an innovative large-scale and cost-effective bioengineering platform has demonstrated effective PD-L1 silencing in NSCLC cells, further expanding the range of RNAi modalities under investigation [15]. This BioRNA construct consists of a transfer RNA (tRNA) scaffold that enhances RNA stability, fused to a pre-miRNA carrier into which the PD-L1-targeting siRNA is inserted. Following intracellular processing by the endogenous RNAi machinery, the construct is converted into a functional siRNA that mediates PTGR of the PD-L1 protein. A few in vivo transfection agents evaluated using BioRNA/siGFP, produced from the same BioRNA/siRNA platform, achieved efficient delivery to both the liver and lungs with well-tolerated safety profiles, which highlights the feasibility and therapeutic potential of advancing BioRNA/siPD-L1 for in vivo applications using optimized delivery platforms [246].

Emerging checkpoint targets such as TIGIT are also under investigation, with the INTASYL^TM^ self-delivering siRNA candidate PH-804 currently undergoing preclinical evaluation in hematological malignancies [16].

In addition to the widely explored siRNA-based therapeutics, two miRNA-based drugs have entered clinical Phase I. MRX34, a miR-34a-5p-based miRNA mimic, delivered by liposomal injection, was a potential first-in-class miRNA-based drug for the treatment of advanced solid tumors (NCT01829971), though major immune-related adverse events led to the termination of the trial [247,254]. Interestingly, the liposomal delivery system employed in MRX34 has demonstrated a favorable safety profile in some other settings, while the immunogenicity of the miR-34a-5p sequence has been proposed as a potential contributing factor [255], in addition to the doses of delivery vehicles and RNA species. Besides the non-specific effects associated with the exposure of double-stranded RNA and infusion-related adverse events, the immune-related toxicities observed with MRX34 may also result from excessive immune activation, as those observed in ICI therapy. This may be attributed to the reported ability of miR-34a-5p to suppress PD-L1 protein expression, which highlights the potential of miR-34a-5p as an immunotherapeutic agent [201,203,204]. The therapeutic efficacy of unmodified miR-34a-5p required repeated administration at relatively high doses because of its susceptibility to nuclease degradation and inefficient tissue-specific targeting. Consistent with these delivery challenges, Phase I pharmacokinetic analyses of MRX34 demonstrated measurable systemic exposure but large interpatient variability and non-linear pharmacokinetic behavior following repeated dosing. Nevertheless, pharmacodynamic analyses confirmed the distribution of miR-34a-5p to tumor tissues and dose-dependent modulation of target gene expression, providing evidence of biological activity in human patients [254]. These findings support the therapeutic promise of miR-34a-5p, while underscoring the need for alternative modalities and delivery platforms of miR-34a-5p to minimize toxicities and improve therapeutic performance [255,256]. Subsequently, a miR-16-5p-based miRNA mimic loaded in EGFR-targeting bacterial EnGeneIC Dream Vector (EDV™) nanocells, namely TargomiRs, has entered Phase I MesomiR (NCT02369198) clinical trial as a 2nd or 3rd line treatment for patients with recurrent MPM [248,249]. Overall, TargomiRs are well-tolerated, though immune-related toxicities are observed [248,249]. Although the contributions of the miR-16-5p mimic and delivery vehicle could not be distinguished, the substantially lower miRNA dose used in the Phase I MesomiR study compared to the MRX34 clinical trial suggests that the miRNA mimic was less likely the primary cause of toxicity [248]. Similar inflammatory toxicity had been previously observed in earlier clinical studies using the same EDV™ carrier to deliver cytostatic drugs, implicating the bacterial-derived carrier as the more probable cause of toxicity [248]. As miR-16-5p is reported to directly silence PD-L1 protein levels and is inversely correlated with PD-L1 expression in tumors from patients with MPM, TargomiRs may exert immunomodulatory effects that contribute to its antitumor activity [197]. However, whether these mechanisms contribute to the inflammatory adverse events observed with TargomiRs remains unclear and warrants further evaluation in later clinical studies.

Collectively, these studies underscore the growing versatility of RNAi technologies in immune-checkpoint modulation and the importance of optimizing delivery strategies to overcome the inherent instability of RNAs, thereby advancing next-generation cancer immunotherapies.

## 7. Conclusions and Perspectives

Cancer immunotherapy by targeting ICPs has significantly transformed the treatment landscape for multiple malignancies. FDA-approved ICIs, targeting CTLA-4, PD-1, and PD-L1, have demonstrated substantial therapeutic benefits across diverse cancer types. Anti-LAG-3 relatlimab became the first FDA-approved antibody against a novel ICP beyond CTLA-4 and PD-1/PD-L1, after demonstrating its clinical benefit in combination with a PD-1 inhibitor. Additional antibodies against emerging ICPs, including TIM-3, LAG-3, and TIGIT, remain under active clinical evaluation. Despite the clinical challenges encountered by these candidates, these novel ICPs remain promising therapeutic targets. Future advances will depend on a deeper understanding of their context-dependent biology, improved patient selection, and the development of rational combination strategies.

Recent advances in RNAi-based therapeutics, particularly siRNA and miRNA agents, may provide promising alternatives for immune checkpoint modulation. Through PTGR, RNAi technologies enable selective and simultaneous targeting of multiple pathways, offering greater flexibility and potential advantages over conventional monoclonal antibody therapies. Increasing evidence from preclinical and clinical studies demonstrates the ability of ICP-targeting RNAs to effectively suppress checkpoint expression and enhance antitumor immune responses across various cancer models.

Despite these promising developments, the clinical application of therapeutic RNAs remains largely limited by the inherent instability of RNA molecules and inefficient cellular uptake. Consequently, the development of efficient delivery systems has become a critical focus in the field. Nanotechnology-based delivery, self-delivering RNA platforms, and biologic RNA modalities are explored to improve RNA pharmacokinetics, tumor targeting, and therapeutic efficacy while maintaining favorable safety profiles.

Future research should focus on optimizing delivery systems, improving tumor-specific targeting, minimizing toxicity, and evaluating combinational strategies integrating RNAi therapeutics with existing immunotherapies. Altogether, RNAi-based therapeutics may represent a promising next-generation strategy capable of expanding and improving the future of cancer immunotherapy.

## Figures and Tables

**Table 1:** FDA-approved ICIs and their key clinical benefits supporting their initial approval.

Drug	Target	Initial Indication	Year of Initial FDA Approval	Comparison Arm	Key Clinical Benefit
Ipilimumab	CTLA-4	Advanced Melanoma	2011	gp100 vaccine	Median OS: 10.1 vs. 6.4 months; ORR: 10.9% vs. 1.5%; DoR ≥ 2 years: 60.0% vs. 0.0%
Tremelimumab + Durvalumab	CTLA-4 + PD-L1	Unresectable HCC	2022	Sorafenib	Median OS: 16.4 vs. 13.8; ORR: 20.1% vs. 5.1%; median DoR: 22.3 vs. 18.4 months
Pembrolizumab	PD-1	Advanced melanoma	2014	Single arm	ORR: 33.0%; median OS: 23 months; DoR > 1 year: 44.0%
Nivolumab	PD-1	Advanced melanoma	2014	Dacarbazine/carboplatin + paclitaxel	ORR: 27.0% vs. 10.0%; median OS: 16.0 vs. 14.0 months
Cemiplimab	PD-1	Advanced cSCC	2018	Single arm	ORR: 47.0%; DoR > 6 months: 57.0%
Dostarlimab	PD-1	Recurrent or advanced dMMR endometrial cancer	2021	Single arm	ORR: 42.3%; median OS: not reached
Retifanlimab	PD-1	Metastatic or recurrent locally advanced Merkel cell carcinoma	2023	Single arm	ORR: 52.3%; DoR ≥ 12 months: 62.0%
Toripalimab	PD-1	Metastatic or recurrent NPC	2023	Single arm	ORR: 20.5%; DoR: 12.8 months; median OS: 17.4 months
Tislelizumab	PD-1	Advanced or metastatic ESCC	2024	Paclitaxel/docetaxel/irinotecan	median OS: 8.6 vs. 6.3 months; ORR: 20.3% vs. 9.8%; median DoR: 7.1 vs. 4.0 months
Atezolizumab	PD-L1	Advanced or metastatic urothelial carcinoma	2016	Single arm	ORR: 27.0% (IC2/3), 18% (IC1/2/3); Median OS: 11.4 months (IC2/3), 8.8 months (IC1/2/3); median DoR: not reached
Avelumab	PD-L1	Metastatic Merkel cell carcinoma	2017	Single arm	median OS: 12.6 months; ORR: 33%; median DoR: 40.5 months
Durvalumab	PD-L1	Advanced or metastatic urothelial carcinoma	2017	Single arm	ORR: 17.8%; median OS: 18.2 months; median DoR: not reached
Relatlimab + Nivolumab	LAG-3, PD-1	Unresectable and metastatic melanoma	2022	Nivolumab	PFS: 10.1 vs. 4.6 months; risk of disease progression/death: −25%

**Table 2:** Human miRNAs shown to regulate ICPs or their ligands with relevance to cancer immunotherapy.

Target ICP	Human miRNA	Context	Reference
CTLA-4	miR-155-5p	human CD8^+^ Treg cells	[189]
	miR-9-5p	human CD8^+^ Treg cells	[189]
	miR-145-5p	human CD4^+^ Treg cells	[190]
	miR-138-5p	CD4^+^ T cells in glioma model	[191]
PD-1	miR-28-5p	mouse T cells in melanoma model	[192]
	miR-374b-5p	cytokine-induced killer cells in liver cancer model	[193]
	miR-138-5p	human CD4^+^ T cells in glioma model	[191]
PD-L1	miR-105-5p	gastric cancer	[194]
	miR-145-5p	bladder cancer	[195]
	miR-502-5p	gastric cancer	[196]
	miR-193a-3p	MPM	[197]
	miR-15a/15b/16-5p	MPM	[197]
	miR-195-5p	diffuse large B-cell lymphoma, TNBC	[198,199]
	miR-497-5p	TNBC, renal cell carcinoma	[199,200]
	miR-200a/b/c-5p	AML, NSCLC	[201,202]
	miR-34a-5p	AML, NSCLC	[201,203,204]
	miR-140-5p	NSCLC, osteosarcoma, cervical cancer	[205,206,207]
	miR-142-5p	cervical cancer, pancreatic cancer	[207,208]
	miR-340-5p	cervical cancer	[207]
	miR-383-5p	cervical cancer	[207]
	miR-148a-3p	colorectal cancer	[209]
	miR-152-3p	gastric cancer	[210]
	miR-17-5p	melanoma	[211]
	let-7-5p	breast cancer, cervical cancer, osteosarcoma, NSCLC, acute monocytic leukemia	[212]
	miR-217-5p	laryngeal cancer	[213]
	miR-138-5p	colorectal cancer, NSCLC	[214,215]
	miR-424-5p	ovarian cancer	[216]
	miR-93-5p	colorectal cancer, TNBC	[217,218]
TIM-3, TIGIT	miR-379-5p	CD8^+^ T cells in breast cancer models	[219]
Gal-9	miR-445-5p	skull base chordoma, colon cancer	[220,221]
	miR-22-3p	liver cancer	[222]
CEACAM1	miR-423-5p	NSCLC	[223]
HMGB1	miR-1284-5p	osteosarcoma	[224]
	miR-142-5p	cervical cancer	[225]
	miR-505-3p	glioma	[226]

**Table 3:** Current ICP-targeting RNAi drug candidates under preclinical and clinical development.

Molecular Targets	RNAi Mechanism	Cancer Type	Stage in Development	Delivery Mechanism	Reference
CTLA-4	siRNA	melanoma	pre-clinical	cationic lipid-assisted PEG-PLA-based nanoparticles	[13]
CTLA-4	siRNA	colon cancer	pre-clinical	INTASYL™ self-delivering siRNA	[232,233,234]
CTLA-4 & STAT3	CTLA-4 aptamer fused to siSTAT3	Melanoma, renal cell carcinoma, lymphoma, and colon cancer	pre-clinical	CTLA-4 internalization from cell surface	[235]
CTLA-4 & PD-1	siRNA	melanoma, colon cancer	pre-clinical	PGLA-PEG based cationic lipid–polymer nanoparticles carrying both CTLA4 aptamer and PD-1 siRNA	[236]
PD-1	siRNA (PH-762)	cutaneous squamous cell carcinoma, melanoma, Merkel cell carcinoma	Phase I	INTASYL™ self-delivering siRNA	[14,237]
PD-L1	siRNA	melanoma	pre-clinical	acid-activatable micelleplexes	[238]
PD-L1	siRNA	melanoma	pre-clinical	pH-responsive micelleplex	[239]
PD-L1 & STAT3	siRNA	melanoma	pre-clinical	Au-CGKRK nanoconjugates	[240]
PD-L1	siRNA	melanoma	pre-clinical	PCL-PEG/PCL-PEI based hybrid micelle	[241]
PD-L1	siRNA	pancreatic cancer	pre-clinical	iron oxide-based magnetic nanoparticles	[242]
PD-L1	siRNA	NSCLC	pre-clinical	peptide-branched polymer conjugate with lipid/siRNA complex	[243]
PD-L1	siRNA	breast cancer	pre-clinical	PEGylated PEI-coated bismuthene	[244]
PD-L1	siRNA	breast cancer	pre-clinical	polycationic carrier camouflaged with macrophage membrane	[245]
PD-L1	BioRNA/siRNA	NSCLC	pre-clinical	in vivo transfection agents	[15,246]
potentially PD-L1	miR-34a-5p-based mimic (MRX34)	advanced solid tumors	Phase I (terminated)	liposome	[247]
potentially PD-L1	miR-16-5p-based mimic (TargomiR)	MPM	Phase I	EGFR-targeting bacterial minicell-derived EVs (EDV™)	[248,249]
TIGIT	siRNA (PH-804)	hematological malignancies	pre-clinical	INTASYL™ self-delivering siRNA	[16]

## Data Availability

No new data were created or analyzed in this study. Data sharing is not applicable to this article.

## Abbreviations

The following abbreviations are used in this manuscript:

AGO1-4	Argonaute 1-4
AML	Acute myeloid leukemia
BioRNA	Biologic RNA
CEACAM-1	Carcinoembryonic antigen cell adhesion molecule-1
cSCC	Cutaneous squamous cell carcinoma
CTLA-4	Cytotoxic T-lymphocyte associated protein-4
DGCR8	DiGeorge critical region 8
dMMR	Mismatch repair deficient
DoR	Duration of response
ESCC	Esophageal squamous cell carcinoma
FcγR	Fcγ Receptor
FDA	The United States Food and Drug Administration
Gal-3	Galectin-3
Gal-9	Galectin-9
HCC	Hepatocellular carcinoma
HMGB1	High-mobility group protein B1
ICI	Immune Checkpoint inhibitor
ICP	Immune checkpoint protein
LAG-3	Lymphocyte activation gene-3
LSECtin	Liver and lymph node sinusoidal endothelial cell C-type lectin
MCC	Merkel cell carcinoma
MHC-I/II	Major histocompatibility complex class-I/II
miRNA	MicroRNA
MPM	Malignant pleural mesothelioma
MRE	MiRNA response elements
mRNA	Messenger RNA
NK	Natural killer
NPC	Nasopharyngeal carcinoma
NSCLC	Non-small cell lung cancer
Nt	Nucleotide
ORR	Overall/objective response rate
OS	Overall survival
PARN	Poly (A)-specific ribonuclease
PCL	Polycaprolactone
PD-1	Programmed cell death protein-1
PD-L1/2	Programmed cell death ligand-1/2
PEG	Polyethylene glycol
PEI	Polyethylenimine
PFS	Progression-free survival
PLA	Poly(_D,L_-lactide)
Pre-miRNA	Precursor miRNA
Pri-miRNA	Primary miRNA
PTGR	Posttranscriptional gene regulation
PtdSer	Phosphatidylserine
Treg	Regulatory T lymphocytes
tRNA	Transfer RNA
RNAi	RNA interference
RISC	RNA-induced silencing complex
SCAC	Squamous cell carcinoma of the anal canal
siRNA	Small interfering RNA
TCR	T cell receptor
Th1/2	T helper 1/2
TIGIT	T cell immunoglobulin and ITIM domain
TIM-3	T cell immunoglobulin and mucin domain-containing-3
TNBC	Triple-negative breast cancer
TRBP	Transactivating response RNA-binding protein
UTR	Untranslated region
